# Drought Responses of Foliar Metabolites in Three Maize Hybrids Differing in Water Stress Tolerance

**DOI:** 10.1371/journal.pone.0077145

**Published:** 2013-10-15

**Authors:** Jinyoung Y. Barnaby, Moon Kim, Gary Bauchan, James Bunce, Vangimalla Reddy, Richard Charles Sicher

**Affiliations:** 1 USDA-ARS, Crop Systems and Global Change Laboratory, Beltsville, Maryland, United States of America; 2 USDA-ARS, Environmental Microbial and Food Safety Laboratory, Beltsville, Maryland, United States of America; 3 USDA-ARS, Soybean Genomics and Improvement Laboratory, Beltsville, Maryland, United States of America; Purdue University, United States of America

## Abstract

Maize (*Zea mays* L.) hybrids varying in drought tolerance were treated with water stress in controlled environments. Experiments were performed during vegetative growth and water was withheld for 19 days beginning 17 days after sowing. Genotypic comparisons used measured changes of leaf water potential or results were expressed by time of treatment. Total dry matter of the drought tolerant hybrid on the final harvest was 53% less than that of the intermediate and susceptible maize hybrids when plants were water sufficient. This showed that maize hybrids selected for extreme drought tolerance possessed a dwarf phenotype that affected soil water contents and leaf water potentials. Changes of shoot and root growth, leaf water potential, net photosynthesis and stomatal conductance in response to the time of water stress treatment were diminished when comparing the drought tolerant to the intermediate or susceptible maize hybrids. Genotypic differences were observed in 26 of 40 total foliar metabolites during water stress treatments. Hierarchical clustering revealed that the tolerant maize hybrid initiated the accumulation of stress related metabolites at higher leaf water potentials than either the susceptible or intermediate hybrids. Opposite results occurred when changes of metabolites in maize leaves were expressed temporally. The above results demonstrated that genotypic differences were readily observed by comparing maize hybrids differing in drought tolerance based on either time of treatment or measured leaf water potential. Current findings provided new and potentially important insights into the mechanisms of drought tolerance in maize.

## Introduction

The production of maize is of global importance because of its high yield potential, its many industrial uses and its suitability as an animal feedstock [1]. Most maize growing areas are rain-fed and the crop is subject to periodic water deficits that diminish yields and decrease economic returns [2,3]. Soil water deficiencies result from insufficient precipitation or from the degradation of natural groundwater sources. Global climate change is predicted to increase ambient temperatures and also the frequency and severity of drought in various growing regions that are highly dependent on maize [1,4]. Plant breeders and major seed companies have developed maize genotypes with enhanced yields in water deficient environments. Phenotypic traits, such as silking, yield, grain number, carbon allocation to roots, leaf rolling and leaf chlorophyll content, were used to select stress tolerant maize germplasm [5]. Successful drought resistant genotypes improved commercial maize yields under water limiting conditions by up to 15% and, importantly, yields under water sufficient conditions were only marginally less than control hybrids [6,7]. Although we know a great deal about the agronomic performance of drought tolerant maize hybrids, much less is known about the biochemical and molecular factors that contribute to desiccation tolerance in these hybrid lines. Plant responses to soil moisture deficits usually include stomatal closure, decreased rates of net CO_2_ assimilation, a shift from shoot to root growth and large scale adjustments of plant metabolism [8-10]. Typically osmolytes, compatible solutes and compounds involved in controlling reactive oxygen species are synthesized in response to drought [11-15]. We recently reported changes of primary metabolism in maize leaves using control and water stressed vegetative plants [16]. In this earlier study about 85% of the major metabolites in maize leaves were impacted by drought and specific metabolites, such as proline and malate, were sensitive indicators of water stress in maize leaves. Prior investigators also have reported quantitative and/or qualitative changes of metabolites from a number of drought tolerant and drought sensitive species in response to soil water deficits [17-20]. As expected, drought tolerant genotypes generally retained water more efficiently, maintained higher *P*_n_ and accumulated more biomass under water limiting conditions than sensitive lines. Temporal changes of stress related metabolites or transcripts between tolerant and susceptible maize hybrids were primarily a response to varying degrees of water stress at a given point in time and this was strongly dependent upon differences in total biomass and leaf area. In the current study, we asked if drought responsive metabolites differed between tolerant and susceptible maize genotypes when the degree of water stress was similar among genotypes. This was accomplished by expressing results for genotypes differing in drought tolerance based on changes of leaf water potential (LWP) and comparing this to results obtained by time of treatment. 

The objective of the current study was to compare responses to water stress of drought tolerant and susceptible maize hybrids during vegetative growth. We hypothesized that leaf water relations, net CO_2_ assimilation rates and foliar metabolite levels would differ between drought resistant and drought susceptible maize hybrids when compared against changes of leaf water potential. We are unaware of any prior study comparing metabolite responses to drought in maize leaves based on values of LWP. 

## Materials and Methods

### Plant materials

Elite hybrid maize germplasm (*Zea mays* L.) with differing drought sensitivity was obtained from Jerron Schmoll, Technical Services Manager, Pioneer Hi-Bred International, Inc., Columbus, OH, USA. The maize hybrids used in this study were 33P84 (drought susceptible control), P0791HR (intermediate drought tolerance) and P1151HR (highly drought tolerant). These are hereafter referred to as the susceptible (S), intermediate (I) and tolerant (T) maize hybrids, respectively. The two drought resistant maize lines were marketed in 2011 as Optimum AQUAmax^TM^ hybrids. All three maize hybrids in this study are transgenic and contain multiple foreign genes that confer resistance to various herbicides and plant pests. However, resistance to water stress was obtained from native drought tolerance genes obtained by marker specific selection and enhanced breeding techniques. 

Maize seedlings were grown in a matching pair of controlled environment chambers (model M-2, Environmental Growth Chamber Corp., Chagrin Falls, OH, USA) essentially as described previously [16]. Prior to planting, seeds of all three hybrids were imbibed over night on wetted filter paper, soaked for 15 min in one-third strength commercial bleach and were washed thoroughly with sterile, deionized water. After surface sterilization multiple seeds were planted in 1.8 dm^3^ plastic pots filled with vermiculite. The air temperature was 27 + 1 °C, the PPFD was 700 + 40 µmol m^-2^ s^-1^, when measured at pot height, and the ambient CO_2_ partial pressure was 39 + 10 Pa. Plants were grown with a 14 h day/10 h night cycle and prior to drought treatments pots were watered to the drip point once daily with a complete mineral nutrient solution containing 14.5 mM total N. Normally, seeds of each genotype were sown in eight pots per treatment and these were thinned to one plant per pot between 5 and 7 days after sowing (DAS). One chamber in each pair was randomly chosen for drought treatment which was imposed by withholding nutrient solution. Chambers chosen to receive water stress treatments were alternated between plantings. Water stress treatments were initiated 17 DAS and were continued for up to 19 d. Relative humidity was not controlled in these experiments but 24 h averages were 65 + 10% prior to initiating drought treatments. Leaf sections of approximately 5 to 10 cm^2^ were removed from the midpoint of the most recent, fully expanded leaf, i.e., normally the 4^th^ leaf from emergence. Samples were harvested between 6 and 8 h after the start of the photoperiod on indicated days using four plants from each drought treatment. Leaf samples were quickly transferred to small envelopes, placed in liquid N_2_ to quench metabolism and stored at -80 °C for up to 1 month prior to analysis without altering the results.

### Metabolite measurements

Stress related foliar metabolites were determined by gas chromatography coupled to mass spectrometry and by Ultra-Performance Liquid Chromatography as described previously [16]. Leaf tissue (~ 30 mg DW) was pulverized in a TissueLyser II bead mill (Qiagen, Valencia, CA, USA) using 2 ml SealRite conical microcentrifuge tubes (USA Scientific, Ocala, FL USA) containing a 6.4 mm diameter ceramic bead (MP Biomedicals, Santa Ana, CA USA). The pulverized tissue was extracted twice with 1.4 ml of 70% aqueous methanol. A mixture of internal standards containing 62.5 nmol of α-aminobutyric acid and 26 μmol of ribitol in a total volume of 45 μl was injected into each sample during the first extraction. The first extraction was heated to 45 °C for 15 min in a water bath. The extracts were combined in a 15 ml Falcon tube and stored at -80 °C for up to 2 weeks. Aliquots of each extract were derivatized for chromatography as described previously [27]. Standard curves were prepared with four point curves using known mixtures of soluble sugars, organic acids and amines. These procedures routinely detected up to 40 total foliar metabolites from maize leaves.

### Gas exchange measurements

Gas exchange parameters were measured during the middle of the light period using the same intact maize leaves that were used for metabolite analyses [16]. Measurements were initiated 17 DAS and were performed at 1 or 2 d intervals throughout the drought treatment. Net CO_2_ and H_2_O vapor exchange rates were determined with a LiCor model 6400 portable Photosynthesis System (Open System 4.0, Li-Cor, Inc., Lincoln, NE). The most recently expanded leaf from a single maize plant was placed in a 1 dm^3^ cuvette and conditions within the cuvette were set to match that of the growth chambers used for plant growth. The PPFD, temperature, CO_2_ partial pressure and relative humidity in the cuvette were 700 μmol m^-2^ s^-1^, 27 °C, 39 Pa and ~ 70%, respectively. Illumination in the cuvette was from a red/blue LED lamp provided by the instrument’s manufacturer. Leakage rates during gas-exchange measurements were determined with an empty cuvette. Gas exchange data were collected using three to five plants from each drought treatment per experiment. Values of *P*_n_ and *g*_*s*_ were calculated by the Photosynthesis System.

### Other methods

Immediately after completing the gas-exchange analyses 6 mm diameter leaf discs were removed from the measured leaves and placed in insulated, sealed cups. Leaf water potential was determined with a model HR-33T dewpoint microvoltmeter after a 1 h incubation period (Wescor, Logan UT, USA). Relative changes of soil water content were determined by measuring pot weights at indicated times and comparing this to pots containing either completely saturated or dried potting material. For biomass determinations plants were separated into root and shoot fractions and these were oven dried at 70 °C for 72 h prior to weighing.

### Statistical comparisons

Results of three completely replicated experiments were combined and significant differences were determined using a two-way Analysis of Variance procedure (StatView, Mountain View, CA). Leaf components were the independent variables and LWP or time of treatment was the dependent variable. For the ANOVA procedure ratios were obtained by dividing values from water insufficient samples with corresponding values for the water sufficient controls. LWP values for individual plant samples were usually divided into six separate zones based on measured leaf water potentials for each maize hybrid. These zones usually ranged from mild (zone 1, from -0.8 to -1.0 MPa) to moderate to severe water stress (zone 6, from -1.6 to -1.8 MPa). Values of LWP more negative than -1.8 were not used because data for the T hybrid line was not available at this water stress level. Hierarchical clusters were constructed as described by Suzuki et al. [28]. Additional technical details were as described previously [16]. 

## Results and Discussion

### Genotypic differences of growth, leaf water potential and leaf gas exchange in response to water stress

**Figure 1:**
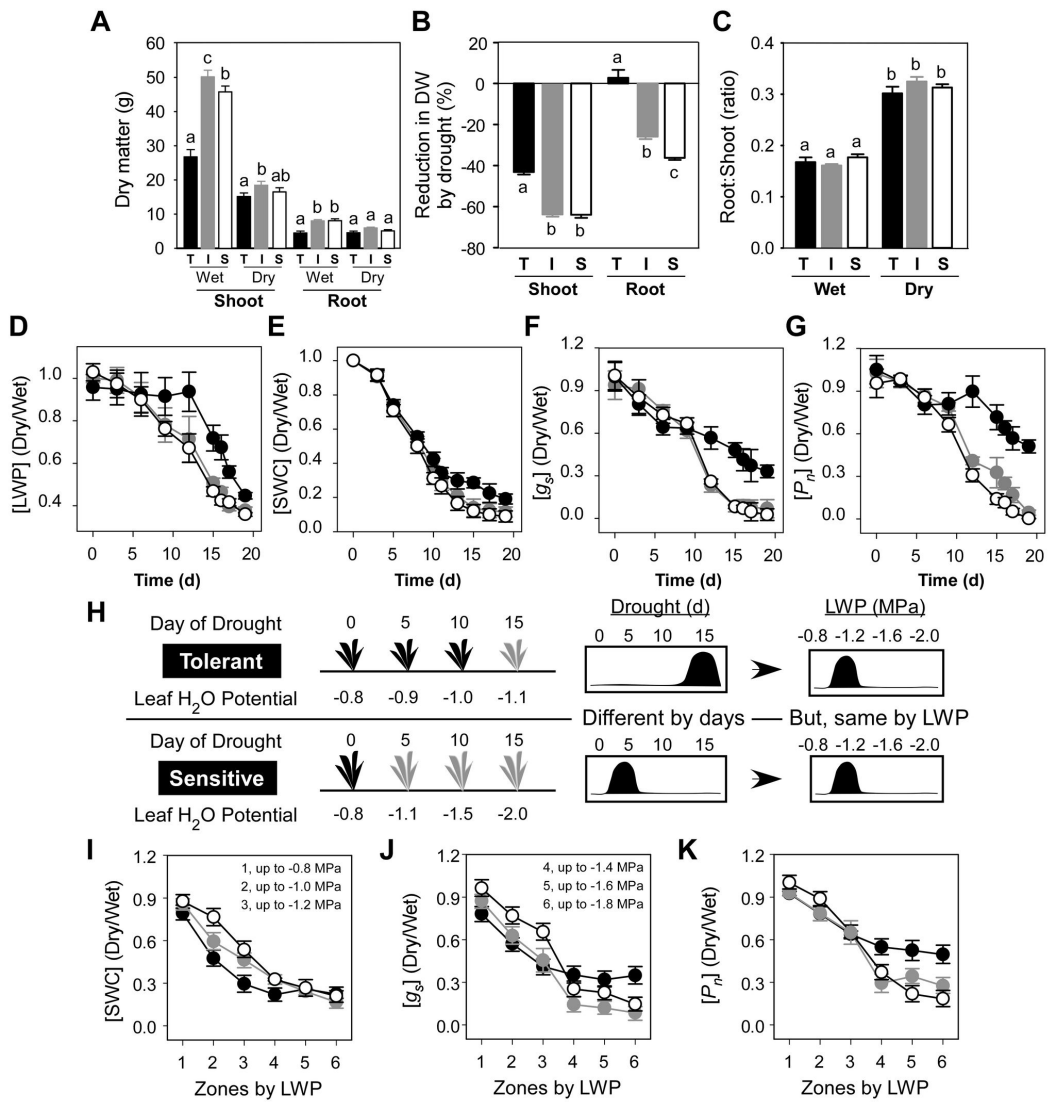
Effects of water stress on maize genotypes differing in drought tolerance. (A) Final shoot and root biomass, (B) percent reduction of shoot and root biomass and, (C) root/shoot ratios for 36 d-old maize seedlings of three maize genotypes differing in drought resistance following 19 d of water stress treatment. (D) Relationship of time of drought treatment and leaf H_2_O potential (LWP) (E) on soil water content (SWC), (F) on stomatal conductance [g_s_] and (G) on single leaf net rates of CO_2_ assimilation [P_n_] are shown. (H) Comparison of a hypothetical plant response to water stress using time of treatment versus changes of leaf water potential (LWP). (I) Relationship of LWP and relative soil water content (SWC), (J) on stomatal conductance [g_s_] and (K) on single leaf net rates of CO_2_ assimilation [P_n_] are shown. Results are for the tolerant (T, black fill), intermediate (I, dark gray fill) and susceptible (S, white fill) maize hybrids. Results for Panels D, E, F, G, I, J and K are shown as ratios obtained for plants from the water insufficient (dry) and water sufficient (wet) treatments. Measured values of LWP for each maize genotype are shown on the horizontal axis (Panels I, J, K). Vertical and horizontal error bars represent SEM (n=8 to 12). Vertical bars of each genotype labeled with a different letter differ at *P* < 0.05 (Panels A, B, C).

Figure 1 shows the effects of water stress on growth, water status and gas exchange rates of the tolerant (T), intermediate (I) and susceptible (S) maize genotypes in response to 19 d of drought treatment. On the final day of treatment, shoot DW of the T maize hybrid was 53.8 and 58.8% less than that of the I and S hybrids in the water sufficient treatment and these values were 82.6 and 86.8% in the water insufficient treatment (Figure 1A). Root DW of the T maize hybrid was 51.8% less than that of the I and S hybrids in the water sufficient treatment, but this changed to 60% versus the I, and 72.6% versus the S hybrid in response to water insufficiency (Figure 1A). On the final harvest, shoot DW of the S, I and T maize hybrids was 63, 63 and 43% lower, respectively, in the water deficient compared to the water sufficient treatments (Figure 1B). Root DW of the S and I maize hybrids also was 25 and 36% less for the water insufficient compared to fully watered plants. Conversely, root DW of the T hybrid increased 2% in response to drought relative to water sufficient plants of the same genotype (Figure 1B). Total DW of the T maize hybrid was 53% less than that of the S and I hybrids under water sufficient conditions, but total biomass accumulation of all three genotypes was similar (*P* > 0.05) under water deficient conditions. Decreased biomass potentially improved the ability of the T hybrid line to efficiently manage leaf water status and to withstand water stress. Interestingly, root/shoot (DW) ratio was similar (*P* > 0.05) among all three genotypes on the final harvest in both the water sufficient and insufficient treatments (Figure 1C). 

As discussed above, plant growth responses of the three maize hybrids to water stress differed and this likely contributed to variations in whole plant water relations. Previous investigations [17-20] reported quantitative differences in metabolites and/or transcripts between drought tolerant and sensitive genotypes based on the duration of water stress treatment. However, if all other parameters remained equal, larger plants of a given species would be expected to consume water more rapidly than smaller plants and this would impact both the timing and extent of drought. Consequently, relative changes of LWP, SWC, *g*_*s*_, and *P*_n_ for the three maize hybrids in response to time of drought treatment were greater (*P* < 0.05) in the S and I than in the comparatively smaller T maize hybrid at later time points (Figure 1D, 1E, 1F, 1G and Table S1). To further test this idea, we compared changes of various physiological parameters for each genotype to ratios of measured values of LWP. When compared using zones of LWP, ratios of SWC for all three genotypes differed in response to mild water stress (*P* ≤ 0.05) (Figure 1I and Table S2) but not when water stress was most severe (i.e., when ratios of SWC were less than 0.3). This important difference was not apparent when relative ratios of SWC were analyzed in response to time of drought treatment (see Figure 1E). The above findings contrasted with gas-exchange measurements shown in Figure 1J and1K. The T hybrid maintained greater *P*_n_ and *g*_*s*_ than either the S or I hybrids on the final measurement. Genotypic differences of *P*_n_ and of *g*_*s*_ in response to changes of LWP were detected for almost every comparison except under mild stress for the S by I hybrids (see ANOVA results in Table S2). Under conditions employed here, the T hybrid did not attain the highly negative LWP values observed for the S or I maize hybrids. Based on the above findings, it was concluded that normalizing the responses of the three maize hybrids to LWP was a potentially important tool for identifying genotypic differences in drought stress tolerance. Another conclusion was that, in general, drought responses of the T maize hybrid differed from the S and I maize hybrids.

### Genotypic differences of foliar metabolites in response to water stress

**Figure 2:**
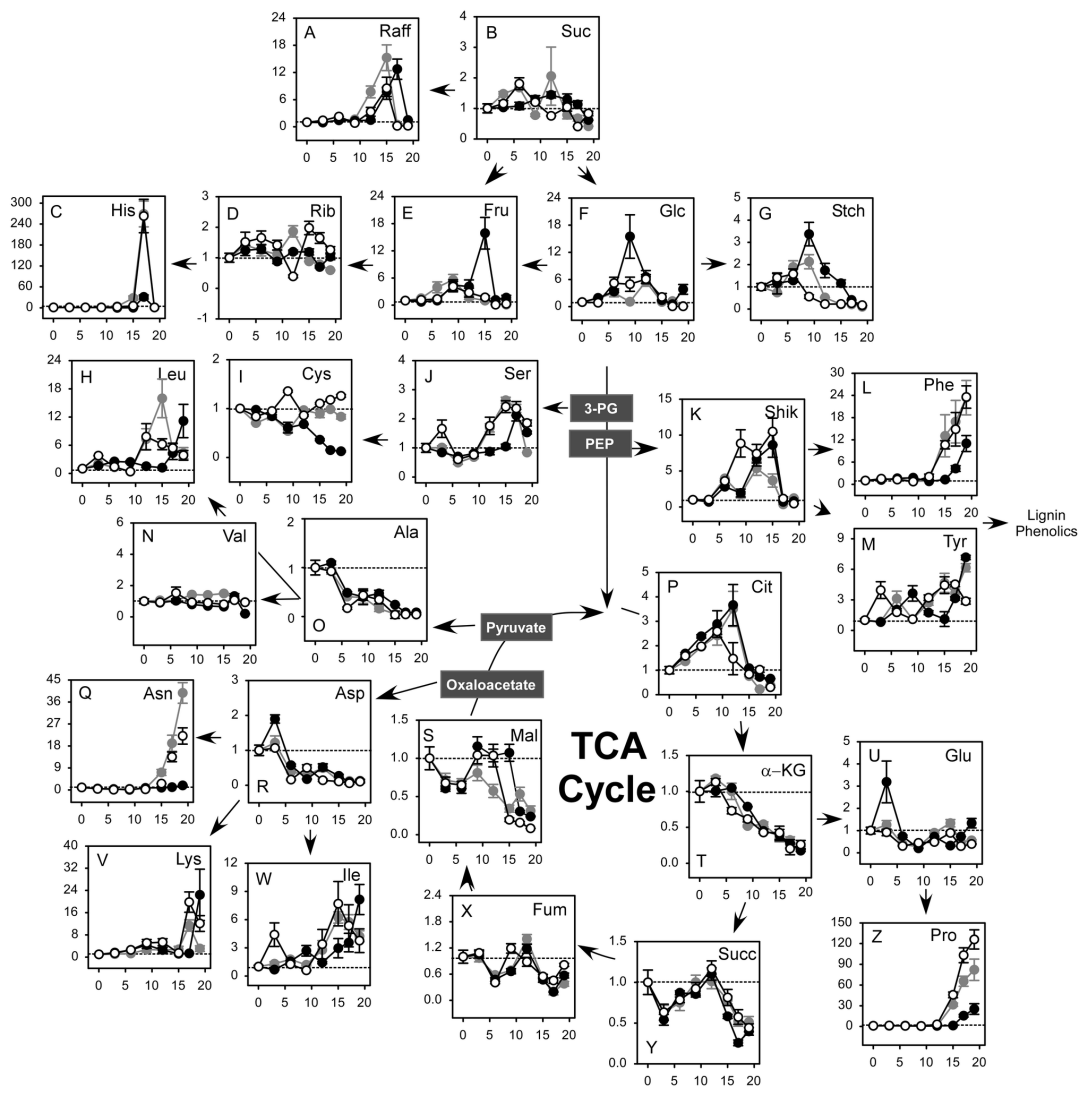
Responses of maize leaf metabolites when plotted as a function of time of drought treatment in three maize genotypes differing in drought tolerance. Results are leaf metabolite ratios (dry/wet) for either the Tolerant (T, black filled), Intermediate (I, dark grey filled) or Susceptible (S, white filled) maize hybrids. Days of drought treatment are shown on the vertical axis. The y-axis shows ratios of leaf metabolites in response to water stress. The dotted line (1 fold) indicates equal metabolite levels in wet and dry samples. Horizontal and vertical error bars represent SEM (n=8 to 12). Abbreviations are for Raff, raffinose; Suc, sucrose; His, histidine; Rib, ribose; Fru, fructose; Glc, glucose; Stch, leaf starch; Leu, leucine; Cys, cysteine; Ser, serine; Shik, shikimate; Phe, phenylalanine; Val, valine; Ala, alanine; Cit, citrate; Tyr, tyrosine; Asn, asparagine; Asp, aspartate; Mal, malate; α-KG, α-ketoglutarate; Glu, glutamate; Lys, lysine; Ile, isoleucine; Fum, fumarate; Succ, succinate; Pro, proline.

Relative increases or decreases of stress responsive metabolites from three maize hybrids differing in drought tolerance were plotted as a function of time of drought treatment as shown in Figure 2. We identified 26 of 40 total maize leaf metabolites that exhibited differences in response to water stress. Qualitative changes of metabolites in maize leaves were in broad agreement with previous drought studies employing maize [16,19]. Briefly, nonstructural carbohydrates, i.e. fructose and glucose, and leaf starch of the T maize line increased transiently due to water stress in the middle stages of the drought treatment (Figure 2). Various aromatic and branched chain amino acids as well as Asn, Lys, and Pro accumulated later in the T hybrid compared to the I or the S hybrids in response to water stress (*P* ≤ 0.05). Several TCA cycle intermediates such as fumarate, succinate, and α-ketoglutarate, and malate, plus Ala and Asp decreased in response to water stress; however, genotypic differences were only observed for malate (*P* ≤ 0.05). Overall, the accumulation of foliar metabolites in response to water stress occurred earlier in the I or S compared to the T hybrid for the most of amino acids except some of the down-regulated amino acids. Genotypic differences in maize leaf metabolites were confirmed using an Analysis of Variance procedure (see Table S1). As discussed above, water loss in response to drought treatment was delayed in the T hybrid, compared to the I and the S hybrids. 

**Figure 3:**
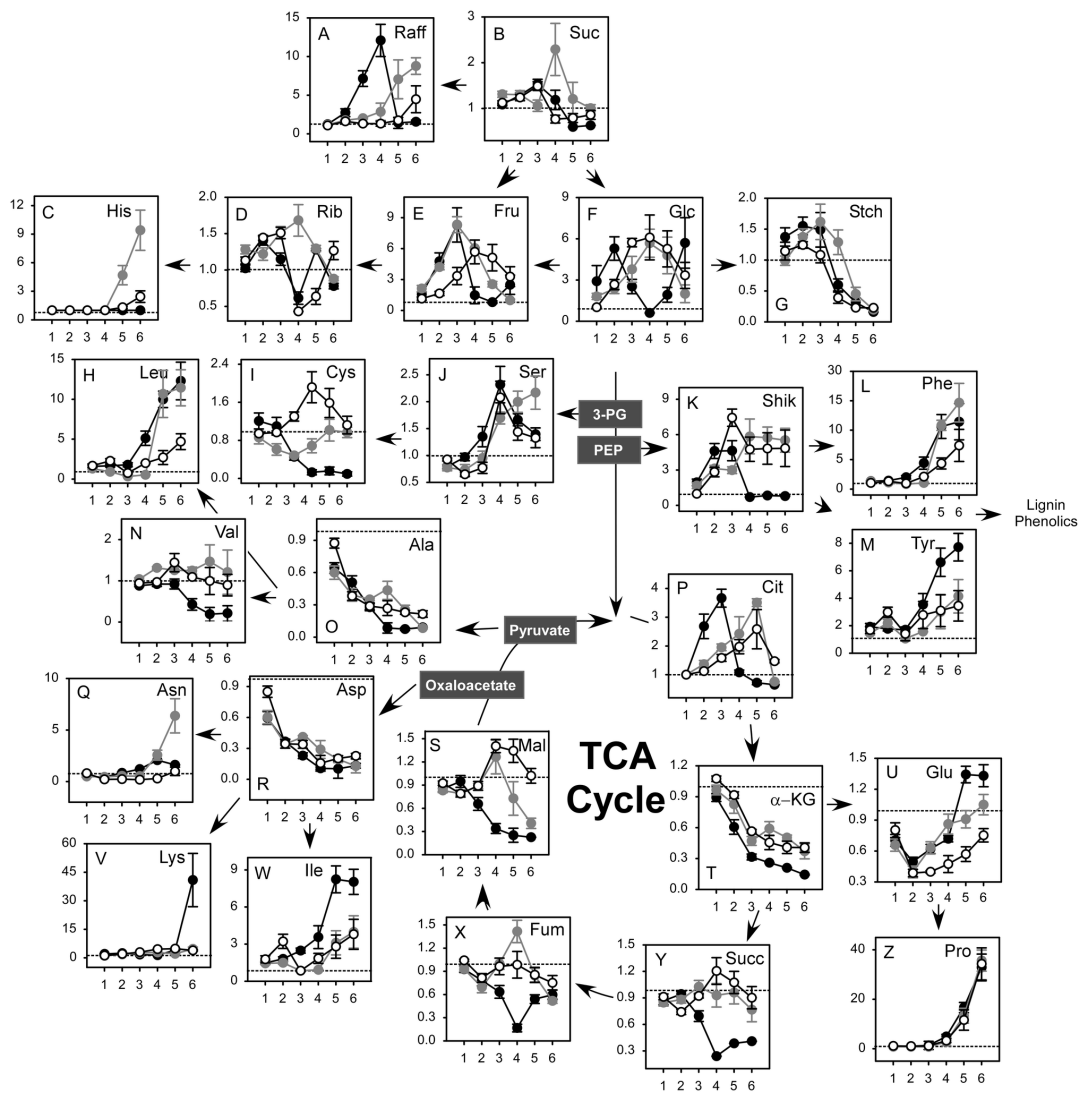
Responses of maize leaf metabolites were plotted as a function of LWP in three maize genotypes differing in drought tolerance. Values are leaf metabolite ratios (dry/wet) for either the Tolerant (T, black filled), Intermediate (I, dark grey filled) or Susceptible (S, white filled) maize hybrids. Measured values of LWP are shown on the horizontal axis. The y-axis shows ratios of leaf metabolites from wet and dry treatments. Other details were as in Figure 2.

To interpret metabolite differences among three maize hybrid lines differing in drought tolerance, relative increases or decreases of stress responsive metabolites also were measured based on changes of LWP as shown in Figure 3. A transient induction of nonstructural carbohydrates occurred in the T hybrid under mild stress (Figure 3A, 3B, 3E, and 3F). This was probably due to a greater reduction of SWC at mild stress levels in the T hybrid compared to the I and the S hybrids (see Figure 1E and 1I). Various aromatic and branched chain amino acids increased and several TCA cycle intermediates plus Ala and Asp decreased in response to water stress. Decreases of several TCA cycle intermediates, including fumarate, succinate, α-ketoglutarate and malate, occurred at higher LWP in the T compared to the S or I hybrids (Figure 3S, 3T, 3X, and 3Y). Similarly, the accumulation of several foliar metabolites occurred at less negative LWP in the T or I compared to the S hybrid. Examples of this were citrate, raffinose, Phe, Tyr, Glu, Lys, Ile, and Leu (Figure 3A, 3H, 3L, 3M, 3U, 3V, and 3W). Genotypic differences in maize leaf metabolites were confirmed using an Analysis of Variance procedure (Table S2). For these calculations measured values of LWP from -0.8 to -1.8 MPa were divided into –three zones corresponding to mild, moderate and severe water stress. This confirmed the above stated conclusion that these metabolites decreased at higher LWP values in the T compared to the S or I hybrids in response to changes of LWP and showed that the detected genotypic differences of metabolite changes in response to water stress were statistically significant. 

Soluble carbohydrates are compatible solutes and osmolytes that have the potential to protect higher plants from abiotic stress [21]. Specific carbohydrates also prevented cellular damage from active oxygen species [13]. In the current study (Figure 3B) and previously [16,22] only small changes of sucrose occurred in maize leaves exposed to water deficits. However, up to 10-15 fold increases of raffinose, glucose and fructose in all three hybrids were observed when compared to at same water stress levels (Figure 3A, 3E and 3F). A substantial accumulation of raffinose, which is an important stress related metabolite in higher plants [23], occurred in all three maize genotypes in the current study. However, maximal raffinose accumulation was observed at more negative LWP values in the S or I compared to the T maize hybrid line. Hexose accumulation also occurred during the mild or moderate stages of water stress in the T hybrid T compared to the S or I hybrids. The above described differences of foliar soluble carbohydrate accumulation potentially contributed to the improved drought tolerance of water stress resistant genotypes of maize by enhancing osmotic protection early in the drought cycle.

Not all metabolites in this study exhibited genotypic differences in response to drought when plotted against LWP. Proline is an important drought-induced metabolite that functions in osmotic regulation and in defense against reactive oxygen species [24-26]. In the current study, Pro increased over 100 fold in response to drought and maximum Pro levels were observed in the S hybrid on the final sampling date (Figure 2Z). However, changes of Pro were similar for all three genotypes when compared against changes of LWP (*P* > 0.05, see Table S2) This indicated that increases of Pro in maize leaves in response to water stress were strongly dependent upon changes of LWP in all three genotypes. Also, decreases of aspartate and alanine in response to severe and very severe drought were similar (*P* > 0.05) among genotypes (Figure 2O, 2R and 3O, 3R). This concurred with our earlier conclusion that Asp and Ala decreased in water stressed maize leaves in response to nitrogen rather than water insufficiency [16]. Overall, the drought tolerant maize hybrid transiently induced hexoses and raffinose, reduced intermediates of the TCA cycle and accumulated stress-related amino acids at greater LWP values than the S or I hybrids when compared against changes of LWP. 

### Hierarchical clustering analysis of foliar metabolites in response to water stress

**Figure 4:**
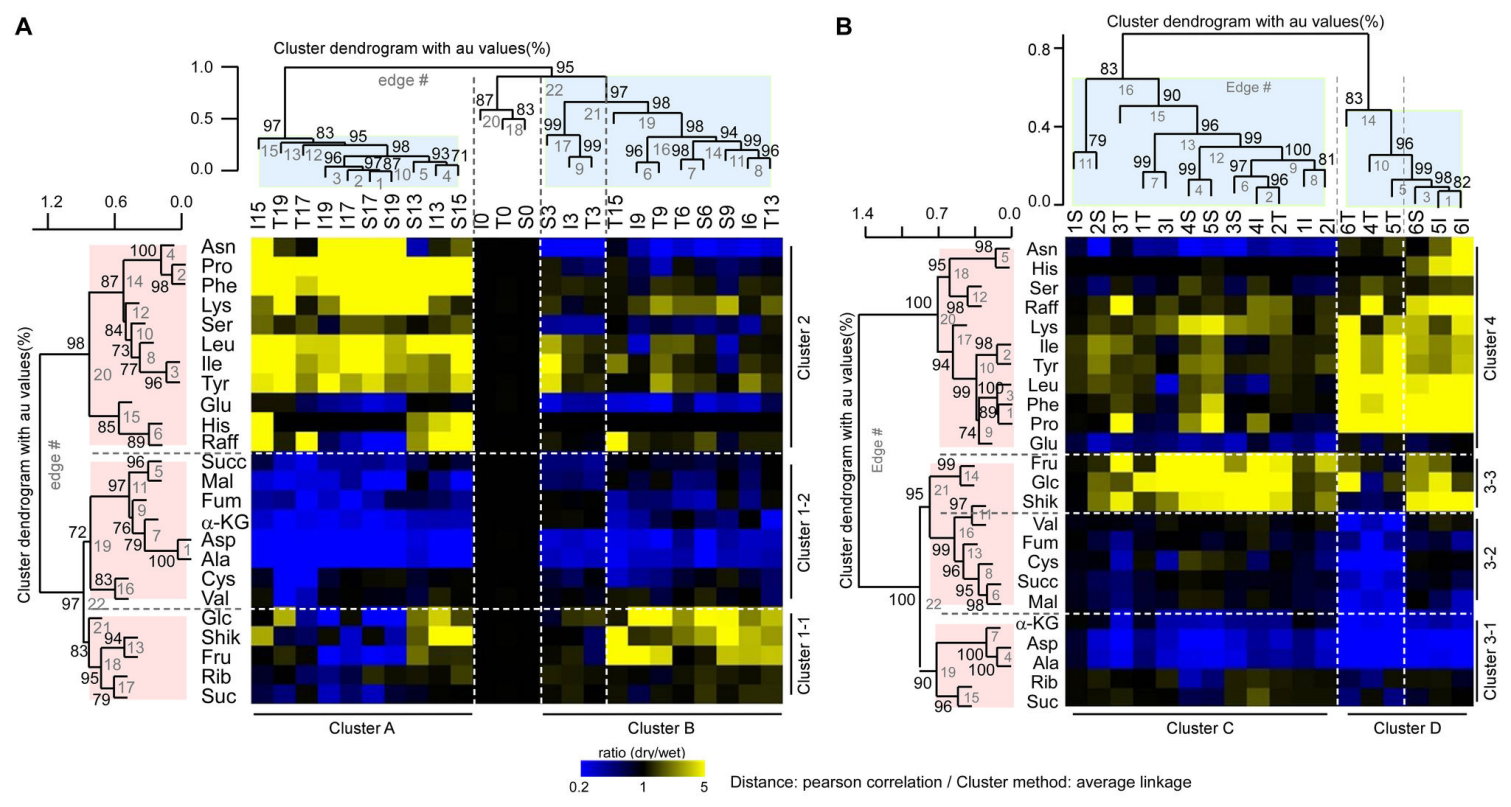
Two-way Hierarchical clustering analysis of metabolites in maize hybrids differing in drought tolerance. Hierarchical clusters (heat maps) of (A) Time of drought treatment (B) Changes of LWP are shown for the tolerant (T), intermediate (I) and sensitive (S) maize hybrid lines. Statistical relationships depicting ratios (dry/wet) of leaf metabolites (vertical axis) in the water sufficient and water insufficient treatments are shown on the vertical axis and six zones of varying LWP [zone 1 (-0.8 to -1.0) through zone 6 (-1.6 to -1.8)] are shown on the horizontal axis (see text for details). Metabolite ratios greater than 1 are in gradations of red up to 5x (see scale bar upper left), ratios that were unchanged are shown in black and ratios less than 1 are in gradations of green. Au indicates approximately unbiased *P*-values (0-100%, with higher numbers denoting greater significance).

Two-way Hierarchical Clustering (HCL) was used to identify relationships among various stress responsive metabolites in three maize hybrids differing in drought tolerance. Metabolite data from all three genotypes were clustered using a Pearson Correlation to generate distance matrices and to assemble the metabolite clusters (labeled with numerals). This was directly compared with water stress clusters (labeled with letters). The heat map in Figure 4A was constructed using time of drought treatment and the one in Figure 4B used zones of LWP. The derived heat maps indicated that there was a strong statistical relationship between Cluster A and Cluster 2 in Figure 4A, and Cluster D and Cluster 4 in Figure 4B. Cluster 2 contained metabolites that accumulated 5 fold or more in response to water stress (colored red). Cluster A included time of drought treatment, i.e., Day 17 and 19 of the T maize hybrid, Day 13 to Day 19 of the I and the S hybrid (Figure 4A). The same metabolites (Cluster 2) were clustered (Cluster 4) with LWP groupings 4 to 6 of the T maize hybrid, groupings 5 and 6 of the I hybrid, and group 6 of the S hybrid (Cluster D) (Figure 4B), which is contrary to the pattern in Figure 4A. This shows that plant water status is an important factor in identifying genotypic differences in drought stress tolerance. 

Three other metabolite associations were evident in the heat map when plotted against change of LWP (Figure 4B). First, fructose, glucose and shikimate were closely associated (Cluster 3-3). These three metabolites among three maize hybrid lines accumulated from mild to severe water stress except the T hybrid at zone 4-6. Second, α-ketoglutarate, aspartate and Ala were present in Cluster 3-1 and were distributed equally in LWP Clusters C and D. These three metabolites decreased (green color) in water stressed maize leaves at low, moderate and severe stages of drought. Third, Val, fumarate, Cys, succinate, and malate (Cluster 3-2) of the T hybrid decreased significantly at moderate and severe water stress levels (LWP zone 4, 5, and 6 of Figure 4B), which was not evident when plotted by time of drought treatment (Figure 4A).

**Figure 5:**
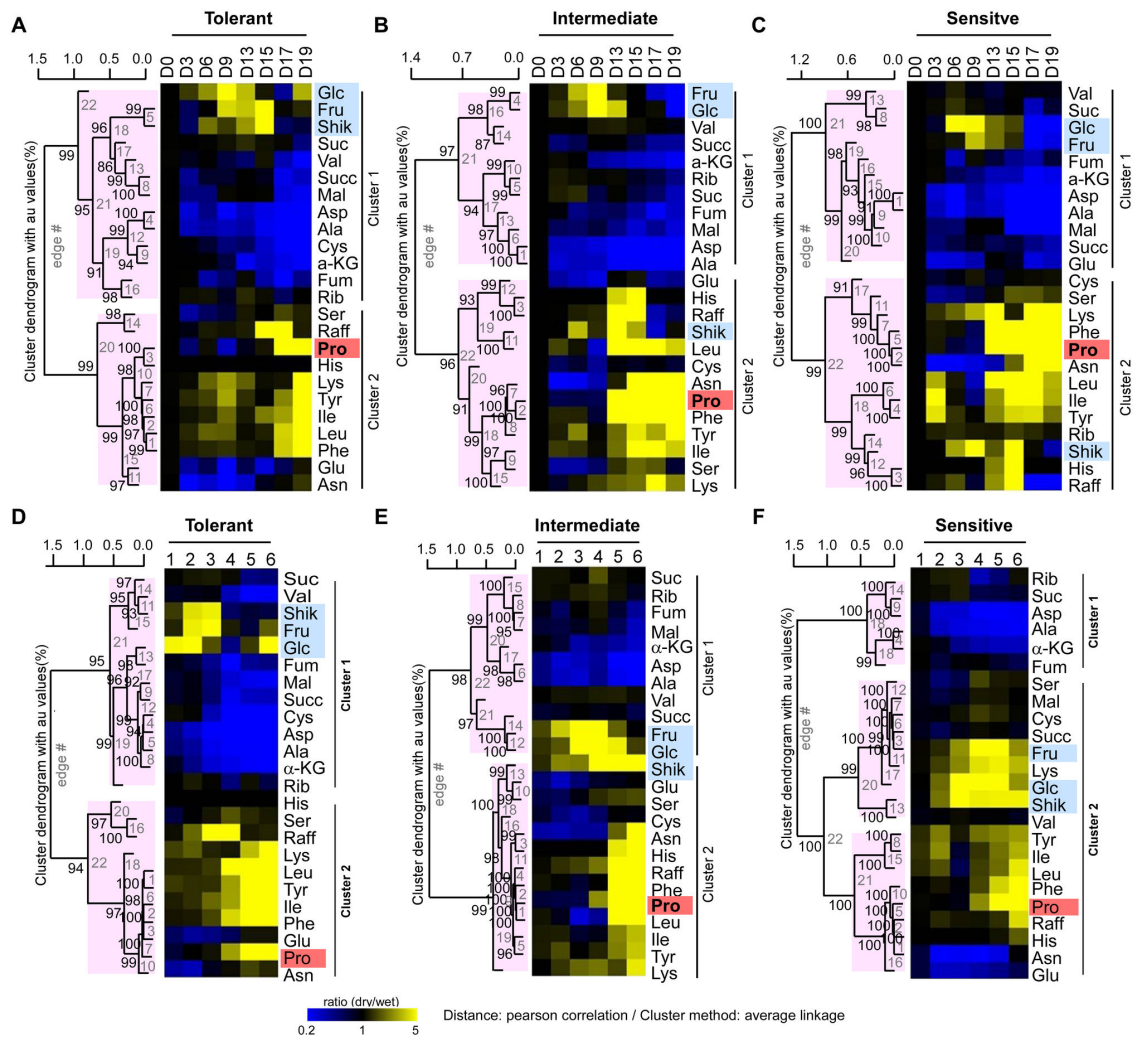
One-way Hierarchical clustering analysis of metabolites in maize hybrids differing in drought tolerance. Hierarchical clusters are shown for metabolite changes in maize leaves in response to drought. Data are for a water stress Tolerant (A, D), Intermediate (B, E) or Susceptible (C, F) maize hybrid. Results were compared based on time of drought treatment (A, B, C) or used changes of LWP (D, E, F). Others details were as in Figure 4.

Responses to water stress were further analyzed using a one-way HCL technique that examined each genotype separately (Figure 5). Again, results were expressed on the basis of time of treatment (Figure 5A, 5B, 5C) or of LWP (Figure 5D, 5E, 5F). Two main metabolite clusters were identified in water stressed maize leaves. Cluster 1 contained metabolites that decreased in response to drought or accumulated under mild water stress. Cluster 2 contained metabolites that accumulated when water stress was moderate to severe. When compared as a function of time of drought treatment, metabolites that accumulated in response to moderate or severe decreases of LWP (Cluster 2) were less numerous in the T hybrid (11 metabolites) than in the S or I hybrid lines (13 metabolites). Accumulation of metabolites more than 5 fold were observed only on day 17 and 19 in the T hybrid while5 fold accumulations of metabolites occurred as early as day 13 in the I and the S hybrid lines (Figure 5A, 5B, 5C). However, when compared against changes of LWP, metabolites in Cluster 2 were less numerous for the T hybrid (11 metabolites) than for the S or I hybrids (18 and 13 metabolites, respectively). This may indicate that these 11 metabolites in the T hybrid line are essential compounds playing an important role in drought tolerance. Another observation found by LWP analysis was that glucose, fructose, and shikimate accumulated in response to mild water stress in the T hybrid, but in response to moderate water stress in the I hybrid, and in the response of moderate and severe water stress in the S hybrid (Figure 5D, 5E, 5F). Overall, when compared using zones of LWP the T hybrid responded to drought earlier (Figure 1I), and drought responsive metabolites increased or decreased at lesser LWP values than either the I or S hybrids (Compare Figure 5D with Figure 5E and 5F). 

### Concluding remarks

The above results demonstrated that the T maize genotype, which was selected for improved yields in water stressed environments, differed during vegetative growth in responses to water stress from the S and I maize hybrids. One potential advantage of the T versus the S or I hybrids was decreased total biomass under water sufficient conditions. The T hybrid also showed lesser changes of *P*_n_ and *g*_*s*_ in response to water stress than the two other hybrids with increased susceptibility to drought. Greater changes of seven leaf metabolites that decreased and eight that accumulated in response to water stress were observed in the T or I compared to the S maize hybrid when the data were plotted against changes of LWP. Hierarchical clustering also revealed various genotypic differences among metabolite responses to mild, moderate and severe drought. The ability to accumulate stress related metabolites in response to small decreases of LWP may have contributed to the enhanced drought tolerance of the T compared to the S or I hybrids. 

## Supporting Information

Table S1 **ANOVA comparisons (tests of pair-wise interactions) for the effects of time of drought treatment on foliar responses of three maize genotypes differing in drought tolerance.** For analysis, day 0 to 6, day 9 to 12, and day 15 to19 were combined, respectively. Genotypes were tolerant (T), intermediate (I) or susceptible (S) to water stress. Metabolite abbreviations were as in Figure 2. *, *P* ≤ 0.05; **, *P* ≤ 0.01; ns, *P* > 0.05.
(DOC)

Table S2 **ANOVA comparisons (tests of pair-wise interactions) for the effects of leaf water potential (LWP) on foliar responses of three maize genotypes differing in drought tolerance.** For analysis, the zone 1 and 2 (mild stress, up to -1.2 MPa), the zone 3 and 4 (moderate stress, from -1.3 to -1.6 MPa), and the zone 5 and 6 (severe water stress, from -1.7 to -2.0 MPa) were combined. Genotypes were tolerant (T), intermediate (I) or susceptible (S) to water stress. Metabolite abbreviations were as in Figure 2. *, *P* ≤ 0.05; **, *P* ≤ 0.01; ns, *P* > 0.05.
(DOC)
