# Supplementary material for: Drought Responses of Foliar Metabolites in Three Maize Hybrids Differing in Water Stress Tolerance
Source: PLoS One. 2013 Oct 15;8(10):e77145. doi: 10.1371/journal.pone.0077145 (PMC3797120; doi:10.1371/journal.pone.0077145)
Supplement: Table S2 — ANOVA comparisons (tests of pair-wise interactions) for the effects of leaf water potential (LWP) on foliar responses of three maize genotypes differing in drought tolerance. For analysis, the zone 1 and 2 (mild stress, up to -1.2 MPa), the zone 3 and 4 (moderate stress, from -1.3 to -1.6 MPa), and the zone 5 and 6 (severe water stress, from -1.7 to -2.0 MPa) were combined. Genotypes were tolerant (T), intermediate (I) or susceptible (S) to water stress. Metabolite abbreviations were as in Figure 2. *, P ≤ 0.05; **, P ≤ 0.01; ns, P > 0.05. (DOC) [file pone.0077145.s002.doc]

Supplementary Table 2. ANOVA comparisons (tests of pair-wise interactions) for the effects of leaf water potential (LWP) on foliar responses of three maize genotypes differing in drought tolerance**.** For analysis, the zone 1 and 2 (mild stress, up to -1.2 MPa), the zone 3 and 4 (moderate stress, from -1.3 to -1.6 MPa), and the zone 5 and 6 (severe water stress, from -1.7 to -2.0 MPa) were combined. Genotypes were tolerant (T), intermediate (I) or susceptible (S) to water stress. Metabolite abbreviations were as in Figure 2. *, *P* ≤ 0.05; **, *P* ≤ 0.01; ns, *P* > 0.05.

|  | LWP | To -1.2 Mpa | | | To -1.6 Mpa | | | To -2.0Mpa | | |
| --- | --- | --- | --- | --- | --- | --- | --- | --- | --- | --- |
|  | Geno | T vs I | I vs S | T vs S | T vs I | I vs S | T vs S | T vs I | I vs S | T vs S |
| Physiol. | SWC | ** | * | ** | * | ** | ** | ns | ns | ns |
|  | A | * | ns | ** | ** | ** | * | ** | * | ** |
|  | gs | ** | ns | ** | ns | ** | ** | ** | * | ** |
| Carbohydrates | Fru | ns | ns | ns | ** | ns | ** | ns | ns | ns |
|  | Glc | ** | ns | ** | ** | ns | ** | * | ** | ns |
|  | Raff | ns | ns | ns | ** | ** | ** | ** | ** | ** |
|  | Rib | ns | ns | ns | ** | ns | ** | ns | * | ns |
|  | Suc | ** | ** | ns | * | ** | ** | * | * | ns |
|  | Stch | ** | ns | ** | ** | ns | ns | ns | ns | ns |
| Organic acids | aKG | ** | ns | ** | ** | ** | ** | * | ns | ** |
|  | Fum | ns | ** | * | ** | ** | ** | ** | ns | ** |
|  | Succ | * | ns | ns | ** | ** | ** | ** | ** | ** |
|  | Mal | ns | ns | ns | ns | ns | ns | ns | ** | ** |
|  | CIt | ** | ns | ** | ** | ** | ** | ** | ** | ** |
|  | Shik | ns | ns | ** | ns | ** | * | ** | ** | ** |
| Amino acids | Pro | ns | ns | ns | ns | ns | ns | ns | ns | ns |
|  | Asp | ns | ns | * | ns | ns | ns | ns | ns | ns |
|  | Ala | ns | ** | ** | ns | ns | * | ns | ns | ns |
|  | Glu | * | ns | ns | ns | ns | ns | ns | ** | ** |
|  | Val | ns | ns | ns | ** | ns | ** | ** | * | ** |
|  | Lys | ns | ns | ns | ns | ns | ns | ** | ns | ** |
|  | His | ns | ns | ns | ns | ns | ns | ** | ** | * |
|  | Asn | ns | ns | ns | ns | ns | ns | ** | ** | ns |
|  | Leu | ns | ns | ns | ns | ** | ** | ** | ** | ** |
|  | Ile | ns | ** | ** | ** | * | ** | ** | ns | ** |
|  | Tyr | ns | * | * | ns | ns | ns | ** | ns | ** |
|  | Phe | ns | ns | ns | ns | ns | ns | ns | ** | * |
|  | Cys | ns | ns | ns | ** | * | ** | ** | ns | ** |
|  | Ser | ns | ** | ** | ** | ** | ** | ** | ** | ** |
